# Supplementary material for: Kaolinite induces rapid authigenic mineralisation in unburied shrimps
Source: Commun Earth Environ. 2025 Jan 3;6(1):4. doi: 10.1038/s43247-024-01983-7 (PMC11698689; doi:10.1038/s43247-024-01983-7)
Supplement: Supplementary file 2 — Supplementary Material [file 43247_2024_1983_MOESM2_ESM.pdf]

Kaolinite induces rapid authigenic mineralization in unburied shrimps

Nora Corthésy[1*], Farid Saleh[1*], Jonathan B. Antcliffe[1], and Allison C. Daley[1]

[1]Institute of Earth Sciences, University of Lausanne, Géopolis, CH-1015 Lausanne, Switzerland

Corresponding authors: N. Corthésy (nora.corthesy@unil.ch)
F. Saleh (farid.nassim.saleh@gmail.com)

Supplementary Methods

Supplementary Table 1. Description of the taphonomic scores (numbered from 0 to 6) of shrimps to quantify the decay stage.

Taphonomic score	*Carapace*	*Appendages*	*Eyes*
0	Transparent	Transparent	Pigmented
1	Getting opaque	Getting opaque	Getting black
2	Formation of white biofilm or black film	Formation of with biofilm or black film	Completely black
3	Cuticle detaching from internal organs	Disarticulated	Detaching
4	Cuticle detached, exposure of gills and internal organs	Detached	Detached
5	Broken, split in pieces, internal organs gone	Broken	Degrading
6	Completely degraded	Completely degraded	Completely degraded

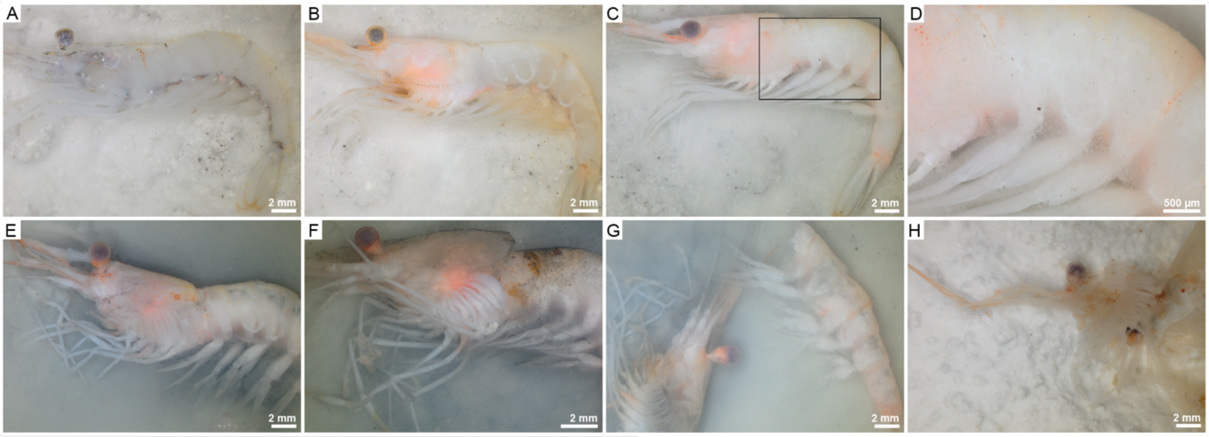

Supplementary Figure 1. Example of the taphonomic scores. (a) Taphonomic score 0 depicts an intact shrimp post-euthanasia. The represented shrimp is decaying on a bentonite bed. (b) Taphonomic score 1 is assigned when the shrimp undergoes color changes and becomes opaque. The shrimp is decaying on a bentonite bed. (c) Taphonomic score 2 is designated when white biofilms appear on the water surface and around the cuticle, as indicated in the rectangle. The shrimp is decaying on a bentonite bed. (d) Zoom in on the framed area of C. (e)

Taphonomic score 3 is given when the carapace begins to detach from the cephalothorax, with the disarticulation of appendages. The shrimp is decaying on a montmorillonite bed. (f) Taphonomic score 4 involves the detached carapace, exposing gills and internal organs, along with appendages detaching from the body. The shrimp is decaying on a montmorillonite bed. (g) Taphonomic score 5 signifies the separation of the cephalothorax from the abdomen, accompanied by highly degraded tissues. The shrimp is decaying on a montmorillonite bed. (h) Taphonomic score 6 represents complete degradation of the shrimp, with only a few remaining tissue fragments. The shrimp is decaying on a montmorillonite bed.

Extended Material and Methods

Model organism: This research took place in the Animal Origins and Morphology Lab (ANOM Lab) at the Institute of Earth Sciences at the University of Lausanne, Switzerland. Freshwater shrimps (*Neocaridina davidi*; 1.5 cm long) and marine shrimps (*Palaemon varians*; 3 cm long) were bred and raised in the Aquarium Research Lab. *N. davidi* were maintained in the same aquarium in water at 25°C and no salinity and were fed with JBL NovoPrawn. *P. varians* were maintained in water with a salinity of 1.024 psu and a temperature of 21°C. They were fed with JBL NovoGranoColor. All the shrimps used in these experiments had reached the adult stage and were randomly chosen from the aquarium but controlled for a target size of 2.5-3cm in length.

Cryogenic Scanning Electron Microscopy: Analysis of three shrimp carcasses post decay were performed at the Electron Microscopy Facility of the University of Lausanne. The three shrimps were placed under the same experimental conditions as the kaolinite systems (5g of kaolinite and 35g of ASW). One of the three shrimps was placed in ASW with salinity of 1.019 psu for ten days, another was placed in ASW with salinity of 1.024 psu for ten days and the third one was placed in ASW with salinity of 1.024 for twenty days. After these periods of decay, the water in the experimental set-ups was removed with sterile pipetting. The shrimps were fixed with Tissue-Tek O.C.T Compound and Colloidal Graphite (Aquadag) on a carbon holder resulting in a low-temperature resistant and conductive paste that allowed the samples to be bonded to the support. The holder was then attached to a transfer rod to place the samples in liquid nitrogen under a vacuum which reached -210°C to vitrify samples and avoid internal ice crystal formation. Once samples were completely frozen, they were placed in the Cryo-SEM chamber (Quorum) and gradually sublimated to remove surface ice. The temperature was then decreased to -140°C. The samples were platinum coated (to 3nm) to increase conductivity and images contrast. SEM images were taken in the presence of secondary (SE) and backscattered (BSE) electron detectors of the Quanta FEG-250 Scanning Electron Microscope. For each shrimp, SEM images, elemental spectra, and an elemental map of eight different random areas of the cephalothorax and the abdomen were acquired using Quanta FEG-250 Scanning Electron Microscope at 10 keV.

Supplementary Results

Detailed statistical analyses

Supplementary Table 2. Contrast analysis of marine shrimps (*P. varians*) to compare the effect of clay minerals (bentonite, kaolinite, montmorillonite, and control) at each timepoint (0-240 hours) of the experiment. Significant p-values are highlighted in bold.

	Estimate	*Standard Error*	*z-ratio*	*p-value*
**Time = 0 hour**				
Control – Bentonite	1.574	1.97	0.797	0.856
Control – Kaolinite	-2.572	9.11	-0.282	0.992
Control – Montmorillonite	-0.718	4.09	-0.175	0.998

Bentonite – Kaolinite	-4.147	9.58	-0.433	0.973
Bentonite – Montmorillonite	-2.292	5.84	-0.392	0.979
Kaolinite – Montmorillonite	1.854	10.79	0.172	0.998
**Time = 24 hours**				
Control – Bentonite	1.348	14.80	0.091	0.999
Control – Kaolinite	0.247	27.79	0.009	1.000
Control – Montmorillonite	-8.843	30.64	-0.289	0.992
Bentonite – Kaolinite	-1.101	20.55	-0.054	0.999
Bentonite – Montmorillonite	-10.191	44.79	-0.228	0.996
Kaolinite – Montmorillonite	-9.090	56.54	-0.161	0.999
**Time = 48 hours**				
Control – Bentonite	1.348	14.80	0.091	0.999
Control – Kaolinite	0.247	27.79	0.009	1.000
Control – Montmorillonite	-8.843	30.64	-0.289	0.992
Bentonite – Kaolinite	-1.101	20.55	-0.054	0.999
Bentonite – Montmorillonite	-10.191	44.79	-0.228	0.996
Kaolinite – Montmorillonite	-9.090	56.54	-0.161	0.999
**Time = 72 hours**				
Control – Bentonite	1.679	1.62	1.036	0.728
Control – Kaolinite	10.131	55.98	0.181	0.998
Control – Montmorillonite	0.614	1.50	0.409	0.977
Bentonite – Kaolinite	8.452	55.98	0.151	0.999
Bentonite – Montmorillonite	-1.065	1.51	-0.704	0.896
Kaolinite – Montmorillonite	-9.517	55.98	-0.170	0.998
**Time = 96 hours**				
Control – Bentonite	3.638	1.51	2.406	0.076
Control – Kaolinite	16.328	14.70	1.111	0.683
Control – Montmorillonite	0.813	1.33	0.611	0.929
Bentonite – Kaolinite	12.689	14.76	0.859	0.826
Bentonite – Montmorillonite	-2.825	1.51	-1.876	0.238
Kaolinite – Montmorillonite	-15.514	14.76	-1.051	0.719
**Time = 120 hours**				
Control – Bentonite	0.677	1.31	0.517	0.955
Control – Kaolinite	5.090	1.55	3.289	**0.006**
Control – Montmorillonite	1.716	1.44	1.191	0.633
Bentonite – Kaolinite	4.413	1.53	2.882	**0.021**
Bentonite – Montmorillonite	1.039	1.43	0.726	0.887
Kaolinite – Montmorillonite	-3.374	1.59	-2.116	0.148
**Time = 144 hours**				
Control – Bentonite	0.607	1.26	0.480	0.964
Control – Kaolinite	5.745	1.54	3.741	**0.001**
Control – Montmorillonite	0.304	1.32	0.230	0.996
Bentonite – Kaolinite	5.138	1.56	3.295	**0.005**
Bentonite – Montmorillonite	-0.303	1.37	-0.221	0.996
Kaolinite – Montmorillonite	-5.441	1.61	-3.380	**0.004**
**Time = 168 hours**				

Control – Bentonite	2.511	1.22	2.053	0.169
Control – Kaolinite	7.649	1.51	5.055	**< 0.001**
Control – Montmorillonite	2.117	1.32	1.607	0.374
Bentonite – Kaolinite	5.138	1.56	3.295	**0.005**
Bentonite – Montmorillonite	-0.394	1.40	-0.281	0.992
Kaolinite – Montmorillonite	-5.532	1.64	-3.383	**0.004**
**Time = 192 hours**				
Control – Bentonite	2.212	1.23	1.793	0.276
Control – Kaolinite	8.451	1.53	5.530	**< 0.001**
Control – Montmorillonite	2.039	1.30	1.574	0.394
Bentonite – Kaolinite	6.239	1.60	3.907	**< 0.001**
Bentonite – Montmorillonite	-0.173	1.40	-0.124	0.999
Kaolinite – Montmorillonite	-6.412	1.64	-3.911	**< 0.001**
**Time = 216 hours**				
Control – Bentonite	3.106	1.32	2.348	0.087
Control – Kaolinite	7.395	1.47	5.022	**< 0.001**
Control – Montmorillonite	2.211	1.32	1.672	0.339
Bentonite – Kaolinite	4.289	1.47	2.924	**0.018**
Bentonite – Montmorillonite	-0.895	1.36	-0.658	0.913
Kaolinite – Montmorillonite	-5.184	1.49	-3.471	**0.003**
**Time = 240 hours**				
Control – Bentonite	2.862	1.31	2.177	0.129
Control – Kaolinite	5.703	1.56	3.657	**0.002**
Control – Montmorillonite	2.650	1.31	2.017	0.182
Bentonite – Kaolinite	2.841	1.52	1.875	0.239
Bentonite – Montmorillonite	-0.212	1.28	-0.165	0.998
Kaolinite – Montmorillonite	-3.053	1.50	-2.031	0.177

Freshwater experiment results

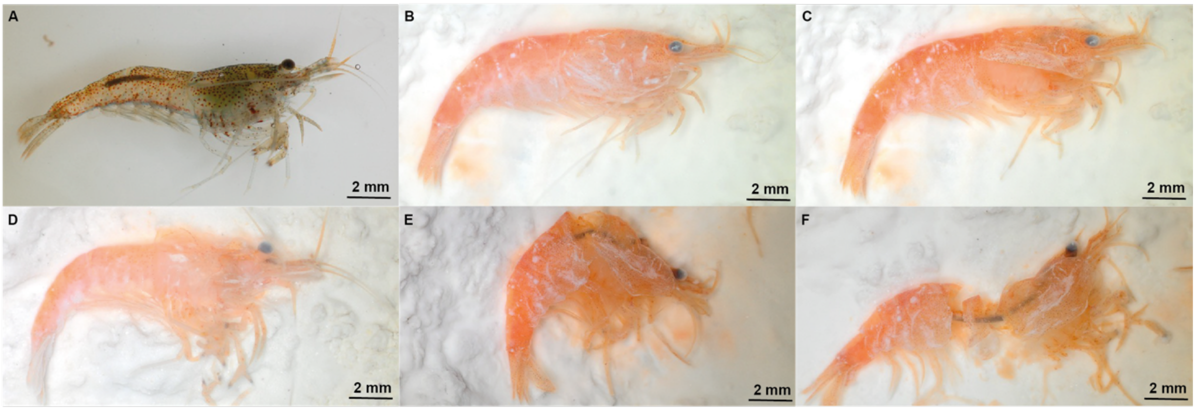

Supplementary Figure 2. Examples of decay stages of freshwater shrimps (*N. davidi*) decaying on kaolinite, bentonite, montmorillonite, and without any sediment. (a) At 0 hours, the shrimps are all intact and transparent (Taphonomic score 0). (b) At 24 hours of decay, the cuticle becomes opaque and pink (Taphonomic score 1). (c) At 48-96 hours, the carapace detaches from the thorax and the abdomen, and gills are exposed (Taphonomic score 3). (d) The carapace is completely detached at 96-144 hours (Taphonomic score 4). (e, f) The cephalothorax and abdomen are split. The body anatomy is destroyed, and internal organs are removed at 144-168 hours (Taphonomic

score 5). The first shrimp (a) is deposited in water without clay. All the other pictured shrimps (b-f) are decaying on a kaolinite bed.

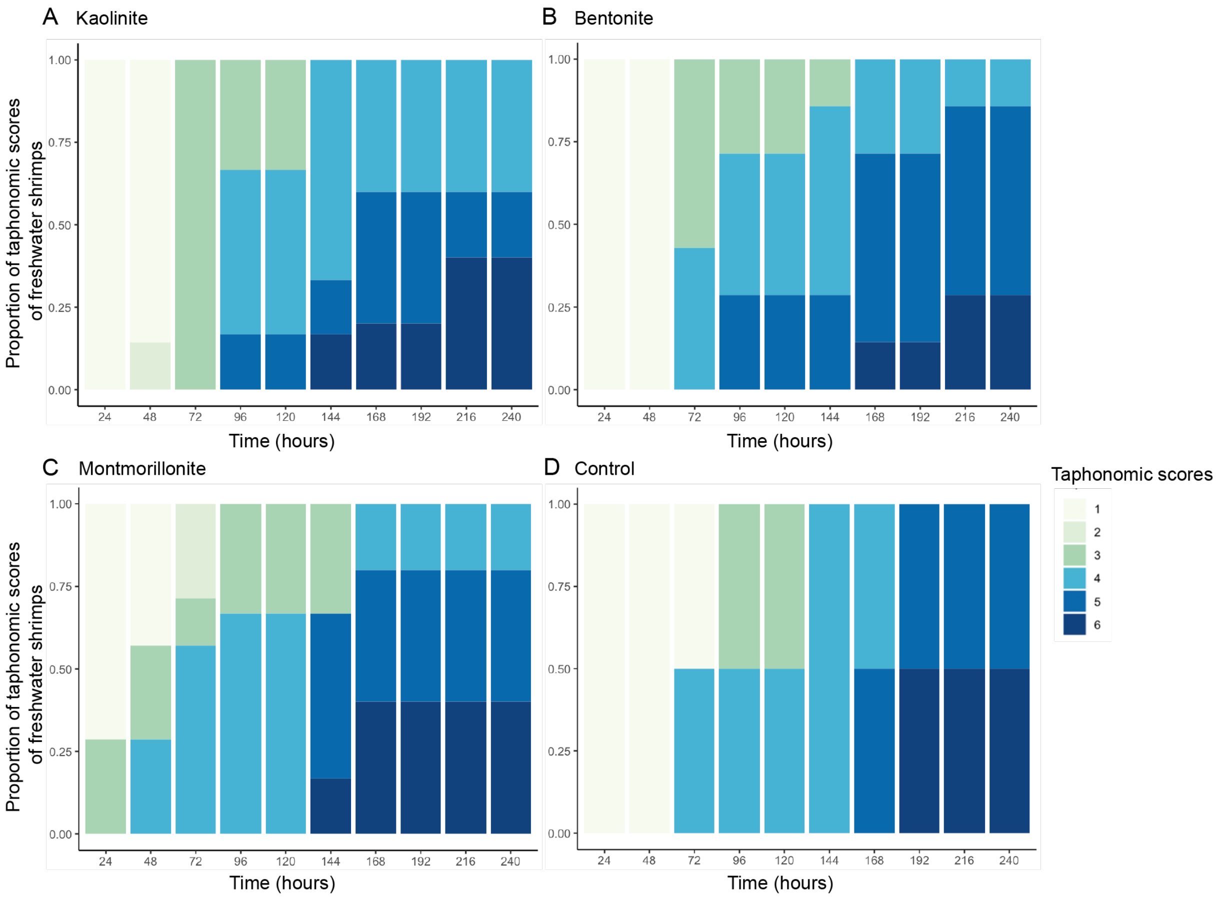

Supplementary Figure 3. Representation of the tissue preservation of decaying freshwater shrimps in each experimental condition in deionized water. Proportion of the taphonomic score according to time (a) in the presence of kaolinite (n = 7), (b) in the presence of bentonite (n = 7), (c) in the presence of montmorillonite (n = 7), and (d) in the absence of sediment (n = 2). Taphonomic scores, quantifying the decay state, are represented by different colors. Darker blue colors indicate more advanced decay.

Detailed statistical analyses

Supplementary Table 3. Contrast analysis of freshwater shrimps (*N. davidi*) to compare the effect of clay minerals at each timepoint (0-240 hours). Significant p-values are highlighted in bold.

	Estimate	*Standard Error*	*z-ratio*	*p-value*
**Time = 0 hour**				
Control – Bentonite	1.826	2.120	0.861	0.825
Control – Kaolinite	0.618	1.173	0.527	0.953
Control – Montmorillonite	1.185	2.900	0.409	0.977
Bentonite – Kaolinite	-1.208	1.192	-1.014	0.742
Bentonite – Montmorillonite	-0.641	4.865	-0.132	0.999
Kaolinite – Montmorillonite	0.567	3.747	0.151	0.999
**Time = 24 hours**				
Control – Bentonite	0.635	23.274	0.027	1.000
Control – Kaolinite	-4.197	19.176	-0.219	0.996
Control – Montmorillonite	-13.617	21.698	-0.628	0.923

Bentonite – Kaolinite	-4.832	4.349	-1.111	0.683
Bentonite – Montmorillonite	-14.252	44.938	-0.317	0.989
Kaolinite – Montmorillonite	-9.420	40.860	-0.231	0.996
**Time = 48 hours**				
Control – Bentonite	-1.008	2.022	-0.498	0.959
Control – Kaolinite	-12.393	1.103	-11.231	**< 0.001**
Control – Montmorillonite	-15.435	1.017	-15.174	**< 0.001**
Bentonite – Kaolinite	-11.385	2.578	-4.417	**0.001**
Bentonite – Montmorillonite	-14.428	2.538	-5.685	**< 0.001**
Kaolinite – Montmorillonite	-3.043	1.383	-2.199	0.123
**Time = 72 hours**				
Control – Bentonite	-1.507	2.041	-0.738	0.882
Control – Kaolinite	-0.290	2.027	-0.143	0.999
Control – Montmorillonite	-1.430	2.069	-0.691	0.900
Bentonite – Kaolinite	1.216	0.984	1.235	0.604
Bentonite – Montmorillonite	0.076	1.040	0.073	0.999
Kaolinite – Montmorillonite	-1.140	1.043	-1.093	0.694
**Time = 96 hours**				
Control – Bentonite	-1.454	1.525	-0.953	0.776
Control – Kaolinite	-0.907	1.533	-0.591	0.935
Control – Montmorillonite	-0.501	1.496	-0.335	0.987
Bentonite – Kaolinite	0.547	1.145	0.478	0.964
Bentonite – Montmorillonite	0.954	1.102	0.866	0.823
Kaolinite – Montmorillonite	0.406	1.115	0.364	0.984
**Time = 120 hours**				
Control – Bentonite	-1.454	1.525	-0.953	0.776
Control – Kaolinite	-0.907	1.533	-0.591	0.935
Control – Montmorillonite	-0.501	1.496	-0.335	0.987
Bentonite – Kaolinite	0.547	1.145	0.478	0.964
Bentonite – Montmorillonite	0.954	1.102	0.865	0.823
Kaolinite – Montmorillonite	0.406	1.115	0.364	0.984
**Time = 144 hours**				
Control – Bentonite	-0.441	1.489	-0.296	0.991
Control – Kaolinite	-1.163	1.515	-0.768	0.869
Control – Montmorillonite	-1.625	1.577	-1.031	0.731
Bentonite – Kaolinite	-0.723	1.086	-0.666	0.910
Bentonite – Montmorillonite	-1.185	1.169	-1.014	0.741
Kaolinite – Montmorillonite	-0.462	1.192	-0.388	0.980
**Time = 168 hours**				
Control – Bentonite	-0.825	1.427	-0.578	0.939
Control – Kaolinite	-0.646	1.510	-0.428	0.974
Control – Montmorillonite	-1.713	1.525	-1.123	0.675
Bentonite – Kaolinite	0.179	1.083	0.165	0.998
Bentonite – Montmorillonite	-0.889	1.098	-0.809	0.850
Kaolinite – Montmorillonite	-1.067	1.207	-0.884	0.813
**Time = 192 hours**				

Control – Bentonite	1.576	1.488	1.059	0.714
Control – Kaolinite	1.755	1.570	1.118	0.679
Control – Montmorillonite	0.687	1.571	0.438	0.972
Bentonite – Kaolinite	0.179	1.083	0.165	0.998
Bentonite – Montmorillonite	-0.889	1.098	-0.809	0.850
Kaolinite – Montmorillonite	-1.067	1.207	-0.884	0.813
**Time = 216 hours**				
Control – Bentonite	0.887	1.485	0.597	0.933
Control – Kaolinite	1.216	1.609	0.756	0.874
Control – Montmorillonite	0.687	1.571	0.438	0.972
Bentonite – Kaolinite	0.329	1.150	0.286	0.992
Bentonite – Montmorillonite	-0.199	1.098	-0.182	0.998
Kaolinite – Montmorillonite	-0.529	1.260	-0.420	0.975
**Time = 240 hours**				
Control – Bentonite	0.887	1.485	0.597	0.933
Control – Kaolinite	1.216	1.609	0.756	0.874
Control – Montmorillonite	0.687	1.571	0.438	0.972
Bentonite – Kaolinite	0.329	1.150	0.286	0.992
Bentonite – Montmorillonite	-0.199	1.098	-0.182	0.998
Kaolinite – Montmorillonite	-0.529	1.260	-0.420	0.975
