# Supplementary material for: Kaolinite induces rapid authigenic mineralisation in unburied shrimps
Source: Commun Earth Environ. 2025 Jan 3;6(1):4. doi: 10.1038/s43247-024-01983-7 (PMC11698689; doi:10.1038/s43247-024-01983-7)
Supplement: Supplementary file 3 — Description of Additional Supplementary Files [file 43247_2024_1983_MOESM3_ESM.docx]

**Description of Additional Supplementary Files**

**File name:** Supplementary Data

**Description:** This file includes the raw data behind the results.

**File name:** Supplementary Material

**Description:** This file includes further details on methods and results.
